# Population variability in social brain morphology for social support, household size and friendship satisfaction

**DOI:** 10.1093/scan/nsaa075

**Published:** 2020-06-08

**Authors:** Arezoo Taebi, Hannah Kiesow, Kai Vogeley, Leonhard Schilbach, Boris C Bernhardt, Danilo Bzdok

**Affiliations:** 1 Department of Psychiatry, Psychotherapy and Psychosomatics, RWTH Aachen University, 52074 Aachen, Germany; 2 Cognitive Neuroscience (INM-3), Institute of Neuroscience and Medicine, Research Center Jülich, 52425 Jülich, Germany; 3 Department of Psychiatry, University Hospital Cologne, 50937 Cologne, Germany; 4 Independent Max Planck Research Group for Social Neuroscience, Max Planck Institute of Psychiatry, 80804 Munich, Germany; 5 International Max Planck Research School for Translational Psychiatry, 80804 Munich, Germany; 6 Department of Psychiatry, Ludwig-Maximilians-Universität, 80336 Munich, Germany; 7 LVR Clinic Düsseldorf, 40629 Düsseldorf, Germany; 8 Multimodal Imaging and Connectome Analysis Lab, McConnell Brain Imaging Centre, Montreal Neurological Institute, McGill University, H3A 0G4 Montreal, Canada; 9 Department of Biomedical Engineering, McConnell Brain Imaging Centre, Montreal Neurological Institute, Faculty of Medicine, McGill University, H3A 0G4 Montreal, Canada; 10 Mila—Quebec Artificial Intelligence Institute, H2S 3H1 Montreal, Canada

**Keywords:** Bayesian hierarchical modeling, big data, social behavior, population neuroscience

## Abstract

The social brain hypothesis proposes that the complexity of human brains has coevolved with increasing complexity of social interactions in primate societies. The present study explored the possible relationships between brain morphology and the richness of more intimate ‘inner’ and wider ‘outer’ social circles by integrating Bayesian hierarchical modeling with a large cohort sample from the UK Biobank resource (*n* = 10 000). In this way, we examined population volume effects in 36 regions of the ‘social brain’, ranging from lower sensory to higher associative cortices. We observed strong volume effects in the visual sensory network for the group of individuals with satisfying friendships. Further, the limbic network displayed several brain regions with substantial volume variations in individuals with a lack of social support. Our population neuroscience approach thus showed that distinct networks of the social brain show different patterns of volume variations linked to the examined social indices.

## Introduction

The importance and complexity of social ties is a central feature of the human species. High-quality social relationships alleviate the risk of adverse mental and physical outcomes (Seeman, 1996). Among the body of studies on this link between sociality and general well-being, a meta-analysis of ~300 000 individuals found that socially well-connected individuals live longer compared to those with weaker social bonds (Holt-Lunstad *et al.,* 2010). Notably, an increase in mortality was related to insufficient social relationships, in terms of both quality and quantity. Such escalated health risk has been found to be more pronounced than factors related to obesity and physical inactivity (Holt-Lunstad *et al.,* 2010). To explain how social relationships may yield beneficial long-term health effects, Eisenberger (2013) proposed a neurocognitive framework, which states that social bonds influence health through its buffering effect on physiological stress responses. Specifically, receiving social support from others would involve brain regions responsive to safety signals, such as the ventromedial prefrontal cortex (vmPFC) (Eisenberger *et al.,* 2011). These brain regions may be involved in inhibiting neural activity in regions involved in threat processing, such as the dorsal anterior cingulate cortex (dACC) and anterior insula (AI) (Eisenberger *et al.,* 2011). Ultimately, modulation of neural activity in threat-related regions has repeatedly been linked to a diminished physiological stress response and may be involved in mediating the ensuing health benefits.

Advantageous health effects, however, are not the only consequences of the state of an individual’s social ties. A wealth of studies suggests a crucial role of close social connections in the way people perceive their surrounding environment. This contention has been supported by functional magnetic resonance imaging (fMRI) studies that investigated the impact of loneliness and social exclusion—two pronounced cases of social disconnection that are manifested in brain response to social cues in the environment. Cacioppo and Hawkley (2009) suggested that lonely individuals are biased to perceive the social world to be more threatening. These individuals demonstrated greater activity in the visual cortex in response to negative social stimuli, suggesting heightened attention to negative social information (Cacioppo *et al.,* 2009). In contrast, individuals in regular contact with close others showed less sensitivity to negative social situations, as evidenced by the reduced neural response observed in the dACC during experiments involving social exclusion (Eisenberger *et al.,* 2007; Masten *et al.,* 2012). In a similar fashion, it has been suggested that socially disconnected individuals display a tendency to avoid negative aspects of the social world as measured by reduced neural activity in regions involved in perspective-taking. Indeed, in an fMRI study conducted by Powers *et al.* (2013), participants that were socially excluded in the experimental paradigm showed decreased neural activity in the dorsomedial prefrontal cortex (dmPFC) to negative social stimuli. Similarly, loneliness has been associated with reduced activity in the temporoparietal junction (TPJ) when exposed to unpleasant social scenes (Cacioppo *et al.,* 2009). As another link between social ties and social information processing in the brain, when viewing pleasant social stimuli, lonely individuals showed a decreased activity in the ventral striatum (Cacioppo *et al.,* 2009). This area includes the reward-related nucleus accumbens. This observation enticed the speculation that lonely individuals find positive social stimuli inherently less desirable or rewarding.

Given the established links between social connections and brain function in previous fMRI experiments, we were wondering whether the form and dynamics of an individual’s closer and wider social network might also be reflected in brain structure. As one early hint, interindividual differences in the size of the broader social network have been observed to show volumetric associations with the orbital prefrontal cortex (Powell *et al.,* 2012), amygdala (Bickart *et al.,* 2011; Kanai *et al.,* 2012b; Von Der Heide *et al.,* 2014) and vmPFC (Lewis *et al.,* 2011). Additionally, loneliness is a general feeling of dissatisfaction with both closer and wider social ties and has recently been found to be related to posterior superior temporal sulcus (pSTS) volume (Kanai *et al.,* 2012a). Further, perceived social support was associated with larger volume in the posterior parts of the posterior cingulate cortex (PCC) (Che *et al.,* 2014) and amygdala (Sato *et al.,* 2016).

However, such previous structural neuroimaging studies on the relation of social bonds have typically relied on small sample sizes and have typically focused on analysis results and interpretation of only a few brain regions. Therefore, in the present study, we were motivated to recognize potential patterns in variation of brain volume related to measures of inner and outer social groups across 36 brain regions at the population level. In this goal to complement and inform existing studies with a population neuroscience perspective, we employed a fully probabilistic hierarchical model on the UK Biobank dataset (Bzdok *et al.,* 2019, 2020), which provides uniformly acquired brain scans and information on social behavior of 10 000 participants. This approach enabled us to probe brain–behavior association in an exploratory fashion, thus avoiding strict a priori assumptions about which putative network in the social brain may be most relevant. To this end, we have enhanced the topographical specificity of our analyses by building them upon the recently established social brain atlas (Alcala-Lopez *et al.,* 2018): four major networks which subdivide a total of 36 target regions. These data-derived regions showed increased neural activation in response to a varied range of social affective tasks in 3972 experiments and several thousand participants. Importantly, this topographical guide enabled our exploratory investigation to examine volume effects from a joint perspective on multiple regions and networks composing the social brain in a principled analysis strategy. As social factors, we centered on three social indices: social support, household size and friendship satisfaction. Markers related to loneliness, social network size and social support provided by the UK Biobank are closely related to the social variables that were previously investigated in brain–behavior studies on the social brain. These factors also reflect three key aspects of benefiting from strong social bonds.

## Material and methods

### Data resources

The UK Biobank (www.ukbiobank.ac.uk) is a prospective epidemiology resource that offers extensive behavioral and demographic assessments, medical and cognitive measures as well as biological samples in a cohort of 500 000 participants recruited from across Great Britain (Sudlow *et al.,* 2015). This openly accessible population dataset provides multimodal brain imaging for 100 000 individuals to be completed only in 2022 (Miller *et al.,* 2016). The present study was based on the data release providing brain imaging recordings from 10 129 individuals to detail the neurobiological properties of the social brain as measured by gray matter (GM) morphology (T1-weighted structural magnetic resonance imaging, MRI). Improving comparability and reproducibility, our study profited from the uniform data preprocessing pipelines (Alfaro-Almagro *et al.,* 2018).

In the present population neuroscience study, we placed emphasis on three social factors that recapitulate important aspects studied in previous social neuroscience experiments: social support, household size and friendship satisfaction. These aspects of social life have been probed in the participants in form of answers to the following questions: to record social support, participants were asked ‘How often are you able to confide in someone close to you?’. The available choices were ‘Almost daily, two to four times a week, About once a week, About once a month, Once every few months, Never or almost never, Do not know, Prefer not to answer’. To assess the household size, participants were asked ‘Including yourself, how many people are living together in your household?’. For friendship satisfaction, in turn, participants were asked ‘In general how satisfied are you with your friendships?’ with the possible answers being ‘Extremely happy, Very happy, Moderately happy, Moderately unhappy, very unhappy, Extremely unhappy, Do not know, Prefer not to answer’. According to these responses to the above questions corresponding to each social factor, the UK Biobank participants were evenly divided along the binary categories (a) ‘with less or more social support’, (b) ‘lives alone or lives with others’ and (c) ‘unhappy or happy with friendships’. To obtain an identical representation across all examined social traits, the quantitative responses have been subject to median splitting.

Our study involved a population sample of 10 129 participants. All of them underwent brain scanning at the same research site (i.e. Cheadle), comprising 47.6% males and 52.4% females. The participants were aged 40–70 years when recruited (Table 1). The present analyses were conducted under UK Biobank application number 25 163. All participants provided informed consent (for details, see http://biobank.ctsu.ox.ac.uk/crystal/field.cgi?id=200).

**Table 1:** Demographic information

	Percent	Mean	SD	Range
Age		55	7.5	40–70
Sex				
Female	52.4			
Male	47.6			
Ethnic background				
British	91.7			
Irish	2.7			
Any other white background	2.3			
Others	3.3			
Household income (£)				
31 000–51 999	27.7			
52 000–100 000	22.6			
18 000–30 999	21.7			
<18 000	12.4			
>100 000	5.4			
Age completed full-time education		17	2.3	5–35
Body mass index (BMI)		26.7	4.3	16.1–63.6

### Brain imaging preprocessing procedures

MRI scanning (3 T Siemens Skyra) was carried out with standard Siemens 32-channel radio-frequency receiver head coils. To protect participant anonymity, imaging data were defaced, and any sensitive information from the header were removed. Automated processing and quality control pipelines were deployed (Alfaro-Almagro *et al.,* 2018). To improve homogeneity, noise was removed by means of 190 sensitivity features. This approach allowed reliably identifying and excluding problematic brain scans, such as due to excessive head motion.

High-resolution T1-weighted images of brain anatomy were acquired using a 3D MPRAGE sequence with 1 mm isotropic resolution. Preprocessing included gradient distortion correction, field of view reduction using the Brain Extraction Tool (Smith, 2002) and linear (Jenkinson and Smith, 2001; Jenkinson *et al.,* 2002) as well as nonlinear registration to MNI152 standard space (Andersson *et al.,* 2007). All image transformations were estimated, combined and applied by a single interpolation step. Tissue-type segmentation into cerebrospinal fluid, GM and white matter was applied using FAST (FMRIB’s Automated Segmentation Tool; Zhang *et al.,* 2001) to generate full bias field corrected images. SIENAX (Smith *et al.,* 2002), in turn, was used to derive volumetric measures normalized for head sizes. The ensuing adjusted volume measurements represented the amount of GM corrected for individual brain sizes.

### Social brain atlas

**Fig. 1:**
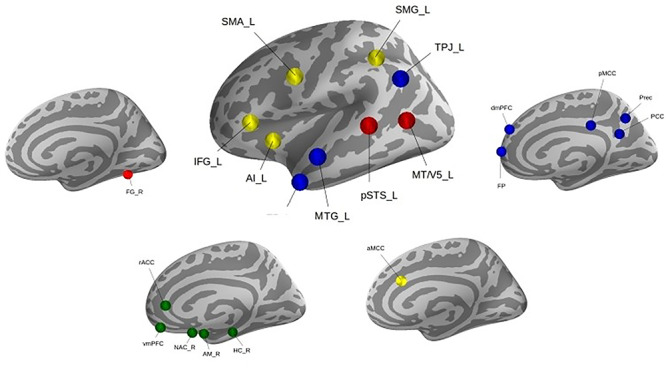
Social brain atlas. Thirty-six brain regions were previously automatically separated into (i) visual sensory network (red), (ii) limbic network (green), (iii) intermediate-level network (yellow) and (iv) highly associative network (blue). For details on the topographical definition, see Alcala-Lopez *et al.* (2018).

Our study benefited from a current best estimate of the ‘social brain’ topography in the human brain (Figure 1). This topographical atlas was derived by quantitative synthesis of a diversity of experimental fMRI findings from 3972 experiments involving several thousand individuals (Alcala-Lopez *et al.,* 2018). Thirty-six volumes of interest were identified with consistent neural activity increases during a diversity of social and affective tasks. Subsequently, these 36 regions were analyzed by meta-analytic connectivity modeling and resting-state whole-brain maps of functional connectivity for each seed. Further, previously conducted clustering analyses showed the target locations to be organized into four functionally coherent networks including: (i) visual sensory network, (ii) limbic network, (iii) intermediate-level network and (iv) highly associative network.

The topographical specificity of the present targeted analyses was thus enhanced by guiding brain volume extraction by the consensus locations of interest. Neurobiologically interpretable measures of GM volume were extracted in the ~10 000 UK Biobank participants by summarizing whole-brain anatomical maps guided by the topographical compartments of the social brain. We applied a smoothing filter of 5 mm FWHM to the participants’ structural brain maps to homogenize local neuroanatomical differences. Local quantities of social brain morphology thus comprised 36 average volume measures for each of the ~10 000 participants. GM volume was extracted in spheres of 5 mm diameter around the consensus location from the atlas, averaging across the voxels belonging to a given target region. Note that using smaller sphere diameters of 2.5 mm or bigger ones of 7.5 mm yielded virtually identical results and led to the same conclusions. This way of engineering distinctly meaningful brain features yielded as many volumetric brain variables per participant as the total number of social brain regions, which have subsequently been *z*-scored by centering to zero mean and unit variance scaled to one. These population estimates of the social brain morphology served as the basis for all subsequent analysis steps.

All of the a priori regions of interest used in this study are available online for transparency and reuse at the data sharing platform NeuroVault (http://neurovault.org/collections/2462/).

### Probabilistic hierarchical regression

To explicitly model the population distribution of brain volume effects linked to specific social factors, we carried out a probabilistic multilevel regression analysis (McElreath, 2015; Bzdok *et al.,* 2019, 2020). We could thus ‘learn from data’ and directly interrogate the population uncertainty intervals of volume effects in their relation to social complexities, rather than restricting attention to differences in mean volume alone. The probability model with a parameter that varies by group followed the following generative process:

$$ L\sim N\left(y,\mathrm{Half}\ \mathrm{Cauchy}\left(0,1\right)\right) $$$$

$$ y\!\!=\!{x}_1^{\ast} \beta{\left(\mathrm{region}\ 1\right)}_{\left[g\right]}+..... +{x}_{36}^{\ast} \beta{\left(\mathrm{region}\ 36\right)}_{\left[g\right]}+{c}_1^{\ast} \beta \left(\mathrm{confound}\ 1\right)+.\dots $$$$

$$ \beta{\left(\mathrm{region}\ 1\right)}_{\left[g\right]}-\beta{\left(\mathrm{lower}\ \mathrm{network}\right)}_{\left[g\right]} $$$$

$$ \beta{\left(\mathrm{region}\ 2\right)}_{\left[g\right]}-\beta{\left(\mathrm{lower}\ \mathrm{network}\right)}_{\left[g\right]} $$$$

$$ \dots \dots $$$$

$$ \beta{\left(\mathrm{region}\ 7\right)}_{\left[g\right]}-\beta{\left(\mathrm{limbic}\ \mathrm{network}\right)}_{\left[g\right]} $$$$

$$ \beta{\left(\mathrm{region}\ 8\right)}_{\left[g\right]}-\beta{\left(\mathrm{limbic}\ \mathrm{network}\right)}_{\left[g\right]} $$$$

$$ \dots \dots $$$$

$$ \beta{\left(\mathrm{region}\ 15\right)}_{\left[g\right]}-\beta{\left(\mathrm{intermediate}\ \mathrm{network}\right)}_{\left[g\right]} $$$$

$$ \beta{\left(\mathrm{region}\ 16\right)}_{\left[g\right]}-\beta{\left(\mathrm{intermediate}\ \mathrm{network}\right)}_{\left[g\right]} $$$$

$$ \dots \dots $$$$

$$ \beta{\left(\mathrm{region}\ 26\right)}_{\left[g\right]}-\beta{\left(\mathrm{higher}\ \mathrm{network}\right)}_{\left[g\right]} $$$$

$$ \beta{\left(\mathrm{region}\ 27\right)}_{\left[g\right]}-\beta{\left(\mathrm{higher}\ \mathrm{network}\right)}_{\left[g\right]} $$$$

$$ \dots \dots $$$$

$$ \beta{\left(\mathrm{region}\ 36\right)}_{\left[g\right]}-\beta{\left(\mathrm{higher}\ \mathrm{network}\right)}_{\left[g\right]} $$$$

$$ \beta \left(\mathrm{lower}\ \mathrm{network}\right)-N\left(0,\mathrm{Half}\ \mathrm{Cauchy}\left(0,1\right)\right) $$$$

$$ \beta \left(\mathrm{limbic}\ \mathrm{network}\right)-N\left(0,\mathrm{Half}\ \mathrm{Cauchy}\left(0,1\right)\right) $$$$

$$ \beta \left(\mathrm{intermediate}\ \mathrm{network}\right)-N\left(0,\mathrm{Half}\ \mathrm{Cauchy}\left(0,1\right)\right) $$$$

$$ \beta \left(\mathrm{higher}\ \mathrm{network}\right)-N\left(0,\mathrm{Half}\ \mathrm{Cauchy}\left(0,1\right)\right) $$$$

where ${x}_i$ denotes the brain volume for all 36 brain regions of the social atlas and $y$ denotes the (*z*-scored) age of the participants. Gaussian-distributed hyper-priors for beta coefficients underlying network-level volume variation jointly inform beta coefficients at the region level for each participant group $g$. Variance that could be explained by the nuisance variables $c$ of body mass and head size was accounted for as potential confounds. The participant groups $g$ indicated stratification of our population sample into male and female with or without presence of a certain social trait. For the example regarding household size, the groups corresponded to [male lives alone], [male lives with others], [female lives alone] and [female lives with others]. This multigroup regression approach also capitalized on the fact that sex and age differences are among the by far largest sources of variability in MRI scans in general (Miller *et al.,* 2016; Ritchie *et al.,* 2018). In this way, we could get the most of our rich sample by borrowing statistical strength between clusters of individuals in our population through interlocking of their model coefficients. Parameters of the within-group regressions, placed at the bottom, were modeled themselves by the hyper-parameters of the across-group regression to pool information across batches of variance components. We could thus provide more coherent and detailed quantitative answers to questions about morphological differentiation of the social brain by a joint model estimation profiting from several sources of population variation.

Approximate posterior inference was achieved by Markov chain Monte Carlo (MCMC) using PyMC3 in Python (https://github.com/pymc-devs/pymc3), which sampled in a random walk toward the target posterior distribution. In 5000 draws, the approximate parameter distributions were improved at each step in the sense of converging to the target distribution. At each step of the MCMC chain, the entire set of parameter values was estimated to be jointly credible given the data. We searched through plausible configurations of parameters as an efficient way of exploring the important part of the parameter space. In particular, we dropped the 4000 first samples from the chain because (1) the chain had probably not yet reached stationarity and (2) this step has reduced dependence on the starting parameter values.

These modeling strategies had at least three advantages (Gelman *et al.,* 2013; McElreath, 2015). First, we could directly quantify the tail area uncertainty of how the region volumes of the social brain vary as a function of sex and social factors. Additionally, the usual problem of multiple comparisons is automatically addressed because hierarchical modeling can find large differences as a byproduct of searching through many parameter constellations (Kruschke, 2010; Gelman *et al.,* 2013). Second, our probabilistic hierarchical regression was aware of the meaningful stratification in our dataset by simultaneously estimating within-group variation and between-group variation from the behavioral and brain data repetitive! Third, appreciating the existing hierarchical structure in our data is known to guard against overfitting to noise and stabilize posterior estimation (Kruschke, 2010).

## Results

Our study investigated the link between key indices of social complexity and population variation in brain morphology in a simultaneous quantitative analysis of four constituent social brain networks and their 36 brain regions (Alcala-Lopez *et al.,* 2018). We have estimated three fully probabilistic hierarchical models (Bzdok *et al.,* 2019, 2020), each targeting one of three social indices: (1) social support, (2) household size and (3) friendship satisfaction. According to these key social factors, the UK Biobank participants were grouped into: (a) ‘male or female with less or more social support’, (b) ‘male or female lives alone or lives with others’, or (c) ‘male or female unhappy or happy with friendships’. In each of our three analyses on a given social index, the resulting model posterior probability distributions are reported as comparisons between these four subgroups for each social trait in a population neuroscience context.

Overall, the results uncovered various network-specific patterns of volume differentiation in relation to the examined social factors. While the visual sensory network displayed large volume effects in both men and women feeling satisfied with their friendships, the intermediate level of neural processing yielded strong volume effects in men with a lack of satisfying friendships. In the limbic network of our social brain atlas, we observed a divergent pattern of volume variations for groups of individuals with a scarcity of supportive relationships. Contrary to the social-dependent structural variations observed in the mentioned networks, the higher-level network only showed a majority of sex-dependent volume effects. In the following, we will go through each of these four neural processing levels and their corresponding volume effects.

### Visual sensory network

**Fig. 2:**
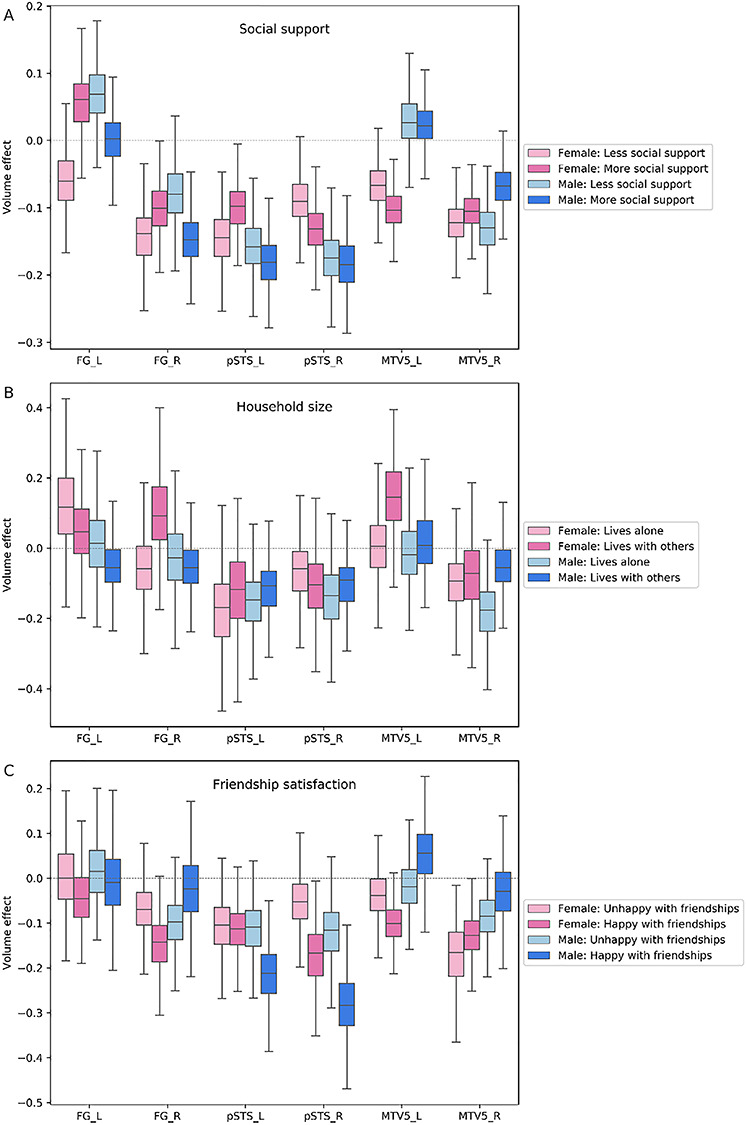
Population volume effects in the visual sensory network of the social brain. Boxplots depict four different subgroups in the context of (A) social support, (B) number of people living in the same household and (C) friendship satisfaction. (A) Men with less social support showed divergent volume effects in the right middle temporal V5 area (MT/V5_R) and left fusiform gyrus (FG_L) compared to men with more social support. (B) Women living with others showed divergent volume effects in the right fusiform gyrus (FG_R) and left middle temporal V5 area (MT/V5_L). (C) Large volume effects were observed in the right and left posterior superior temporal sulcus (pSTS_R, pSTS_L) and left middle temporal V5 area (MT/V5_L) in men and in the right posterior superior temporal sulcus (pSTS_R), right fusiform gyrus (FG_R) and left middle temporal V5 area (MT/V5_L) in women with satisfying friendships. These analyses were conducted in the whole social brain, of which we show obtained marginal posterior distributions for the visual sensory network.

At the lowest hierarchical level of neural processing, regions of the visual sensory network, including the pSTS, fusiform gyrus (FG) and V5 area in the posterior middle temporal gyrus (MT/V5), showed volume effects in at least one of the three examined social indices (Figure 2). Specifically, we observed a divergent trend of volume variation in individuals with the feeling of being satisfied with their friendships. These individuals displayed strong volume effects in the pSTS, right FG and left MT/V5 across examined age groups [pSTS_R/male, posterior mean = −0.282 and 95% highest posterior density interval (HPDI) = −0.154/−0.429; pSTS_L/male, posterior mean = −0.218 (−0.081/−0.358); pSTS_R/female, posterior mean = −0.174 (−0.062/−0.301); FG_R/female, posterior mean = −0.150 (−0.040/−0.284); MT/V5_L/female, posterior mean = −0.102 (−0.014/−0.197); MT/V5_L/male, posterior mean = 0.059 (−0.060/0.191)]. The HPDI indicates the dispersion of 95% certainty that the ‘true’ volume effect for that particular region and examined social trait lies within these boundaries. Moreover, women sharing their homes with others showed volume effects in the left MT/V5 and right FG [MT/V5_L, posterior mean = 0.147 (−0.032/0.336); FG_R, posterior mean = 0.102 (−0.086/0.321)]. In contrast to female-specific volume effects observed in the context of household size, in men, we found volume effects in the right MT/V5 and left FG for those who lack supportive social bonds with close others, compared to men with close social ties [MT/V5_R: posterior mean = −0.133 (−0.069/−0.205); FG_L, posterior mean = 0.066 (−0.026/0.152)].

Summing up our findings in the visual sensory network, the volume effects in this network substantially diverged in the subsets of examined individuals with satisfying friendships. Contrary to the pronounced volume variations in relation to friendship satisfaction, we observed only minor effects in the visual sensory network volume in the context of household size and social support.

### Limbic network

**Fig. 3:**
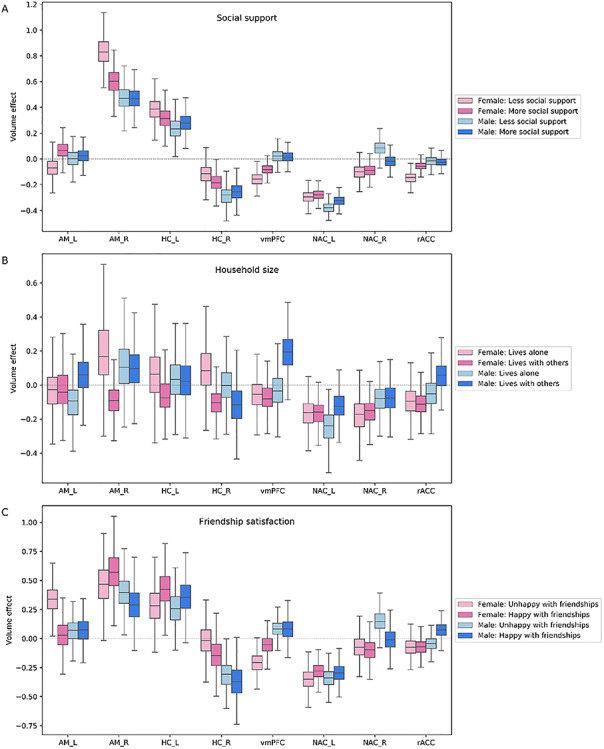
Population volume effects in the limbic network of the social brain. Boxplots depict four different subgroups in the context of (A) social support, (B) number of people living in the same household and (C) friendship satisfaction. (A) Large volume effects were observed in the amygdala (AM), ventromedial prefrontal cortex (vmPFC) and rostral anterior cingulate cortex (rACC) in women and in the right nucleus accumbens (NAC_R) in men with less social support. (B) Volume effects were incongruent in the right amygdala (AM_R) in women and in the ventromedial prefrontal cortex (vmPFC) in men living with others. (C) Volume effects were uncovered in the left amygdala (AM_L) and ventromedial prefrontal cortex (vmPFC) in women and in the right nucleus accumbens (NAC_R) in men who are not happy with their friendships. These analyses were conducted in the whole social brain, of which we show obtained marginal posterior distributions for the limbic network.

We observed incongruent volume effects in the limbic network for groups of individuals with insufficient close social bonds (Figure 3). Women with a lack of close others to confide with exhibited large volume effects in the vmPFC, rostral anterior cingulate cortex (rACC) as well as a prominent volume effect in the amygdala (AM) across examined age groups [vmPFC, posterior mean = −0.156 (−0.059/−0.252); rACC, posterior mean = −0.148 (−0.062/−0.228); AM_R, posterior mean = 0.832 (0.620/1.060); AM_L, posterior mean = −0.073 (0.085/−0.223)]. In a similar fashion, men with less frequent close social interactions showed a notable volume effect in the right nucleus accumbens (NAC) [posterior mean = 0.075 (−0.034/0.172)]. Analogous to the observed volume effects linked to the scarcity of social support, women unsatisfied with their friendships showed strong volume effects in the left AM and vmPFC [AM_L: posterior mean = 0.336 (0.101/0.553); vmPFC, posterior mean = −0.207 (−0.049/−0.367)]. In the same vein, men feeling unhappy with their friendships exhibited a large volume effect in the right NAC [posterior mean = 0.152 (−0.008/0.306)]. Finally, for a subset of individuals living with others at home, we observed volume effects for men in the vmPFC and for women in the right AM [vmPFC, posterior mean = 0.195 (−0.018/0.410); AM_R, posterior mean = −0.085 (0.160/−0.346)].

Summing up our results on the limbic network, at the population level, region volumes deviated to a larger extent in individuals with a scarcity of a stronger close social circle. The lack of supportive relationships and friendship satisfaction was reflected in large volume effects in this network. However, the limbic network also yielded a slight structural effect in individuals living with others.

### Intermediate-level network

**Fig. 4:**
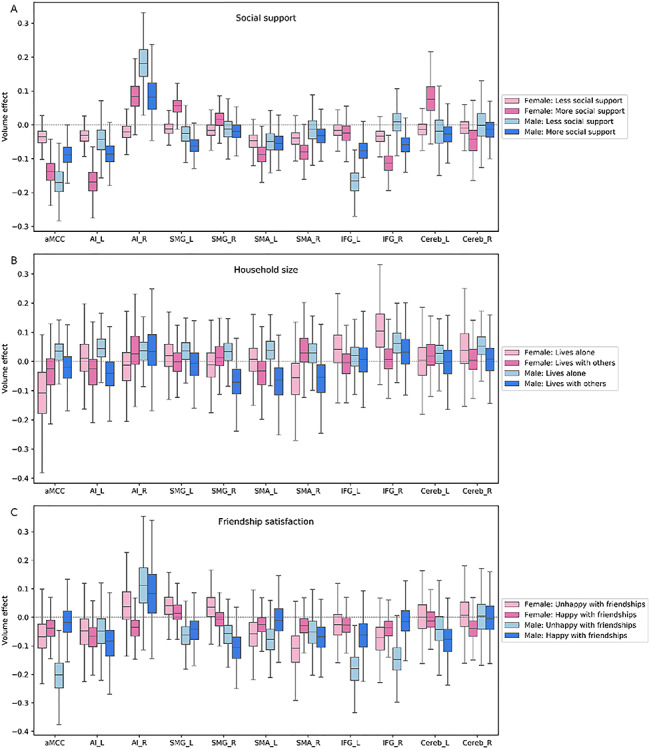
Population volume effects in the intermediate-level network of the social brain. Boxplots depict four different subgroups in the context of (A) social support, (B) number of people living in the same household and (C) friendship satisfaction. (A) Volume effects were incongruent in the anterior midcingulate cortex (aMCC), left anterior insula (AI_L), left supramarginal gyrus (SMG_L), right supplementary motor area (SMA_R), right inferior frontal gyrus (IFG_R) and left cerebellum (Cereb_L) in women with more social support and in the anterior midcingulate cortex (aMCC), right anterior insula (AI_R) and left inferior frontal gyrus (IFG_L) in men with less social support. (B) Men living with others displayed volume effects in the right supramarginal gyrus (SMG_R) and left supplementary motor area (SMA_L) compared to men living alone. (C) Men who are not happy with their friendships showed large volume effects in the anterior midcingulate cortex (aMCC) and inferior frontal gyrus (IFG). These analyses were conducted in the whole social brain, of which we show obtained marginal posterior distributions for the intermediate network.

We observed large volume effects at the intermediate level of neural processing for the group of men unhappy with their friendships (Figure 4). Examining the interindividual variability in social behavior showed pronounced volume effects in the anterior midcingulate cortex (aMCC) and inferior frontal gyrus (IFG) for unhappy men across examined age groups [aMCC, posterior mean = −0.198 (−0.080/−0.323); IFG_L, posterior mean = −0.180 (−0.071/−0.296); IFG_R, posterior mean = −0.147 (−0.045/−0.262)]. Similarly, we observed large volume effects in our analysis pertaining to the amount of social support. However, benefiting from social support appeared to affect men and women in different ways. While women with high social support yielded large volume effects in the intermediate-level regions, men with less frequent social support showed large volume effects in this network [left anterior insula (AI_L)/female, posterior mean = −0.168 (−0.087/−0.250); aMCC/female, posterior mean = −0.137 (−0.063/−0.210); IFG_R/female, posterior mean = −0.114 (−0.050/−0.175); right supplementary motor area (SMA_R)/female, posterior mean = −0.083 (−0.024/−0.143); left cerebellum (Cereb_L)/female, posterior mean = 0.077 (−0.020/0.183); left supramarginal gyrus (SMG_L)/female, posterior mean = 0.055 (0.007/0.095); AI_R/male, posterior mean = 0.182 (0.051/0.289); aMCC/male, posterior mean = −0.172 (−0.100/−0.253); IFG_L/male, posterior mean = −0.171 (−0.102/−0.244)]. Lastly, the right SMG and left SMA, in the intermediate-level network, showed volume effects in men living with others compared to those living alone [SMG_R, posterior mean = −0.074 (0.037/−0.213); SMA_L, posterior mean = −0.072 (0.066/−0.215)].

Summing up our findings on the intermediate level of the neural processing hierarchy, volume effects were particularly incongruent in the context of friendship satisfaction and social support. Further, the amount of social support was only linked to a sex-dependent pattern of volume variations in this network. In contrast to the large volume effects found in the intermediate network for friendship satisfaction and social support, household size was associated with slight volume effects in the intermediate level network.

### Higher associative network

**Fig. 5:**
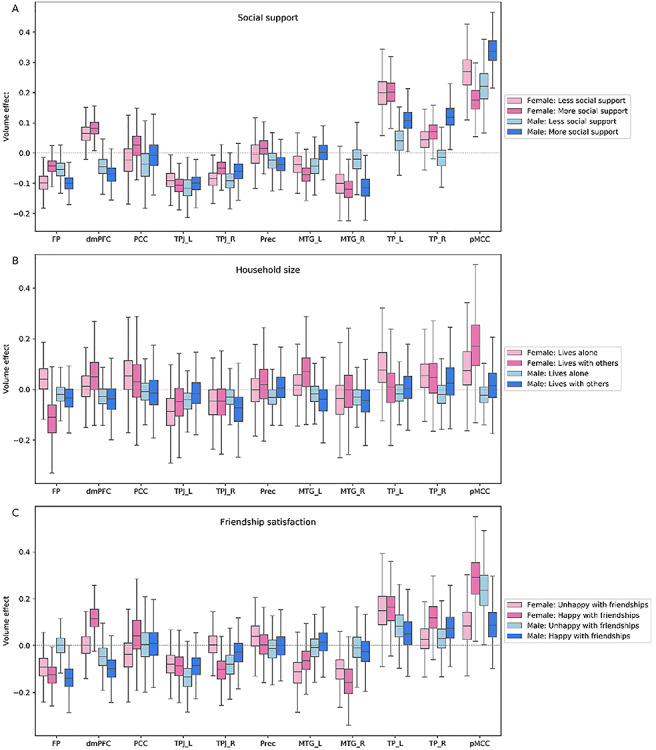
Population volume effects in the higher associative network of the social brain. Boxplots depict four different subgroups in the context of (A) social support, (B) number of people living in the same household and (C) friendship satisfaction. (A) Volume effects were revealed in the frontal pole (FP), right middle temporal gyrus (MTG_R), right and left temporal pole (TP_R, TP_L) and posterior mid-cingulate cortex (pMCC) in men with more social support and in the frontal pole (FP) and posterior mid-cingulate cortex (pMCC) in women with less social support. (B) Household size analysis showed a volume effect in the frontal pole (FP) for women living with others. (C) The volume effects were incongruent in the dorsomedial prefrontal cortex (dmPFC), right temporoparietal junction (TPJ_R) and posterior mid-cingulate cortex (pMCC) in women with happy friendships and in posterior mid-cingulate cortex (pMCC) for men with unhappy and in frontal pole (FP) for men with happy friendships. These analyses were conducted in the whole social brain, of which we show obtained marginal posterior distributions for the higher associative network.

The higher-level network mostly featured a number of sex-dependent volume effects, rather than social-dependent effects of current primary interest (Figure 5). Women, irrespective of the amount of received social support and their friendship satisfaction, tended to be associated with large volume effects in the left temporal pole (TP), dmPFC and right middle temporal gyrus (MTG) across examined age groups [TP_L/females-more social support, posterior mean = 0.201 (0.123/0.273); TP_L/females-less social support, posterior mean = 0.197 (0.109/0.297); dmPFC/females-more social support, posterior mean = 0.084 (0.032/0.137); dmPFC/females-less social support, posterior mean = 0.066 (+0.001/+0.127); MTG_R/females-happy friendship, posterior mean = −0.157 (−0.028/−0.318); MTG_R/females-unhappy friendship, posterior mean = −0.101 (0.032/−0.228)].

Moreover, in this higher-level network, we observed distinct patterns of volume effects for men *vs* women linked to their amount of received social support and friendship satisfaction. While men with sufficient amount of social support showed large volume effects in the posterior mid-cingulate cortex (pMCC), TP, right MTG and frontal pole (FP), we observed volume effects in the pMCC and FP for women with a lack of close social bonds [pMCC/male-more social support, posterior mean = 0.345 (0.249/0.444); TP_R/male-more social support, posterior mean = 0.117 (0.047/0.201); MTG_R/male-more social support, posterior mean = −0.116 (−0.029/−0.188); TP_L/male-more social support: posterior mean = 0.111 (0.028/0.186), FP/male-more social support, posterior mean = −0.102 (−0.046/−0.152); pMCC/female-less social support, posterior mean = 0.262 (0.148/0.371); FP/female-less social support, posterior mean = −0.097 (−0.042/−0.159)]. In a similar vein, women with happy and men with unhappy friendships displayed volume effects in the pMCC [pMCC/female-happy friendship, posterior mean = 0.290 (0.107/0.489); pMCC/male-unhappy friendship, posterior mean = 0.241 (0.062/0.435)]. In addition to the abovementioned sex-dependent trends of volume variations, our result uncovered volume effects in this network linked to friendship satisfaction and household size in both women and men [dmPFC/female-happy friendship, posterior mean = 0.119 (0.033/0.236); TPJ_R/female-happy friendship, posterior mean = −0.103 (0.005/−0.223); FP/male-happy friendship, posterior mean = −0.142 (0.238/−0.030); FP/female-live with others, posterior mean = −0.118 (0.027/−0.289)].

To sum up, we observed large volume effects at the higher level of the processing hierarchy linked to individual differences in benefiting from close social circle. However, the observed links were mostly related to sex differences *per se*. For instance, men receiving social support from supportive interactions yielded volume effects on this network. In contrast, women with a lack of such social interactions showed the social–brain morphology relationship.

## Discussion

Previous studies have highlighted the manifestations of interindividual differences in social interactions on various aspects of the human brain. To complement these previous studies of often small samples with a population neuroscience perspective, we examined the possible relationship between brain volume variation and individual differences in key factors of the closer and wider social environment: social support, household size and friendship satisfaction.

We show that the regions with large volume effects in our study replicate the ones which have been found to be associated with one’s social relationships in previous studies. Specifically, we found substantial structural variations in the regions involved in safety and threat processing, associated with the amount of received social support. This finding is in-line with Eisenberger’s framework on the link between social support and health (Eisenberger, 2013). More importantly, at the level of coherent social networks, individual differences in the considered social indices are linked to divergent volume variations in the social brain in a network-specific manner. We observed notable volume effects in the visual sensory network, associated with friendship satisfaction. In the limbic network, structural variations were mostly observed in relation to supportive relationships. Further, our result showed that men with a lack of satisfying friendships yielded large volume effects in the intermediate-level network. Lastly, individual differences in the considered social dimensions were linked to the structure of higher level regions in a sex-specific manner. It is important to note that in contrast to the pronounced volume variations observed in the context of social support and friendship satisfaction, household size was not associated with less strong volume effects in the social brain.

The lowest level of the neural processing hierarchy yielded substantial volume variations especially in the participants feeling satisfied with their friendships. Indeed, previous studies have emphasized the crucial role of the pSTS, FG and MT/V5 in social information processing such as face perception (Haxby *et al.,* 2000; O'Toole *et al.,* 2002). In general, face perception is thought to rely on invariant and changeable aspects of the face (Haxby *et al.,* 2000). While the FG has been often found to be involved in the processing of invariant aspects of facial features and face identity (Haxby *et al.,* 2000), the pSTS and MT/V5 play a role in the processing of more dynamic facial signals such as facial expressions and movements (Haxby *et al.,* 2000; O'Toole *et al.,* 2002). Thus, our result regarding the link between being satisfied with friendships and greater volume variations in these regions may suggest distinct processing of social cues, such as for other individuals’ faces. This view is consistent with previous studies, which have examined the structural and functional correlates of loneliness, as feeling less satisfied with social relationships (Peplau, 1982). A voxel-based morphometry study (Kanai *et al.,* 2012a) reported that loneliness was associated with reduced GM volume in the pSTS. The authors proposed that the observed relationship between perceived loneliness and the pSTS volume was mediated by deficits in processing eye gaze information. In a similar way, an fMRI study (Cacioppo *et al.,* 2009) demonstrated that loneliness was related to the heightened neural activity in the visual areas when encountering negative social stimuli compared to negative unsocial ones. Taken together, one speculation to explain the present results is that being part of a friendship circle is accompanied by more frequent social contact. Hence, over time, this regular exposure to social signals and decoding these environmental cues may lead to volume adaptations in the visual sensory network. As another, but not mutually exclusive interpretation, the large volume effects found in the visual sensory network may reflect a distinct processing disposition for reading facial cues, which promotes maintaining one’s friendships.

The limbic system that was part of our analysis was the next higher processing level of the social brain hierarchy. In the present study, the volume of the examined limbic regions manifested divergent variations among individuals with a lack of social support and satisfying friendship bonds. Specifically, in our study, an experience of insufficient close social contacts was linked to large volume effects in the vmPFC, rACC and amygdala for women, yet in the NAC for men. The observed link appears to be related to findings of previous structural MRI studies investigating these regions in closely related social variables. In a voxel-based morphometry study, Lewis *et al.* (2011) found a prominent association of GM volume of the vmPFC and both mentalizing competence and social network size. These authors argued that vmPFC morphology measures might be a reflection of the processing power that underlies understanding the mental states of others, a social skill with crucial impact on social interaction dynamics with others. In the same vein, a voxel-based morphometry study (Lebreton *et al.,* 2009) suggested that the GM density in the NAC is closely linked to the sensitivity toward social rewards. Moreover, functional studies showed that the NAC activity was associated with self-disclosure (Tamir and Mitchell, 2012) and feeling understood by others (Morelli *et al.,* 2014). These previous studies point to the idea that sharing personal thoughts and feelings and in turn being understood yield some form of intrinsic reward. The prospect of such gratifications may motivate people to start engaging in and maintaining social relationships with peers.

Similarly, in various studies, the volume of the amygdala has been linked to social network size (Bickart *et al.,* 2011; Von Der Heide *et al.,* 2014) and perceived social support (Sato *et al.,* 2016), suggesting a key role for the amygdala in social life (Bickart *et al.,* 2014). Moreover, concordant with our study, the frequency of benefiting from social support was associated with interindividual differences in amygdala volume (Sherman *et al.,* 2016). The result from this last study is particularly relevant, considering that social support was measured in an analogous fashion to our study. Participants were asked to report how often they received social support over the past 12 months. Also similar to our participants’ age, their sample included adults between 60 and 78 years old, allowing for an accumulation of age-dependent effects.

We were therefore tempted to speculate that a lifestyle with infrequent contacts with others might affect the mentalizing capacity (Atique *et al.,* 2011), social reward processing (Rademacher *et al.,* 2010) and processing of important socioemotional cues from the environment (Etkin *et al.,* 2006; Adolphs, 2010), accompanied by volume adaptations in the limbic network. Conversely, it is also possible that individual differences in these social skills and their neural substrates may restrict the amount of actively manageable social relationships. Moreover, analogous to the social support, friendship quality was manifested in divergent patterns of volume variations in the vmPFC and amygdala for women and the NAC for men in the limbic network. This view concurs with previous functional MRI studies, suggesting that interacting with friends can be considered as rewarding and involves reward-related regions such as the NAC and vmPFC (Guroglu *et al.,* 2008; Wagner *et al.,* 2015).

The intermediate network regions, in turn, yielded volume variations in the aMCC and IFG for the male subgroup with a lack of pleasing friendships. This set of regions has been implicated in cognitive reappraisal (Ochsner and Gross, 2005; Buhle *et al.,* 2014), an ability to reinterpret an emotionally arousing situation in a way that alters its emotional outcome. It is noteworthy in this context that individuals with infrequent use of cognitive reappraisal strategies were found to report higher levels of loneliness (Kearns and Creaven, 2017). In a structural MRI study, Giuliani *et al.* (2011) found that more frequent use of cognitive reappraisal strategies was associated with larger volume in the aMCC. This invites the speculation that our findings may imply that men who are not satisfied with their friendships experience negative emotions. In these situations, cognitive reappraisal strategies can help people downregulate negative emotions (Ochsner and Gross, 2005). Thus, it is possible that divergent volume variations in these regions impact the individuals’ ability of using cognitive reappraisal strategies. Such performance differences could in turn lead the individual to associate more negative feelings to friendship. Moreover, previous studies established the role of the aMCC in response to the adverse social circumstances which convey social disconnection (Eisenberger *et al.,* 2003, 2007, 2011; Masten *et al.,* 2012). This entices us to speculate that feeling unhappy with friendships in our male participants may be processed as feeling socially disconnected and therefore links to this particular system of the human social brain.

Lastly, the sex-specific nature of the here described brain–behavior correspondences was specifically observed in intermediate- and high-level networks. For instance, in higher associative network, our male subgroup with supportive relationships demonstrated strong brain association, while the lack of social support was linked to volume effects in our female participants. Thus, future social neuroscience studies should be alerted to possible sex effects on behavior and brain measurements. Note that the present study is subject to the same interpretational limitations as other neuroscience findings that link phenotypical differences between individuals to their brain basis measured by structural MRI. While in some brain regions, larger GM volume has been associated with increased cognitive performance, other studies showed brain regions to feature a negative association between volume and behavioral outcome (Kanai and Rees, 2011). As such, present structural MRI findings should be taken with caution when speculating about potential behavioral consequences. As a last limitation of our study, future research should take into account the impact of socioeconomic status in our study. This aspect of diversity in human populations has been shown by some previous studies to potentially play a role in the brain systems linked to social cognition (Muscatell, 2018).

## Concluding remarks

Previous neuroimaging research established robust links between interindividual differences in social connections and the anatomy of the human social brain. We detail previous evidence by deploying a fully probabilistic approach (Bzdok *et al.,* 2019, 2020), guided by a recently available social brain atlas, in a large human cohort. Our population findings suggest that maintaining close relationships, indexed by supportive and friendship circle, may be reflected in long-term plasticity effects in the social brain as network-specific population volume effects. Additionally, our evidence is consistent with the idea that social interaction reverberates in higher-level brain structures differently in men and women.

## Funding

D.B. was supported by the Healthy Brains Healthy Lives initiative (Canada First Research Excellence Fund) and by the CIFAR Artificial Intelligence Chairs program (Canada Institute for Advanced Research). D.B. was also funded by the Deutsche Forschungsgemeinschaft (DFG, BZ2/2–1, BZ2/3–1 and BZ2/4–1; International Research Training Group IRTG2150), Amazon AWS Research Grant (2016 and 2017) as well as the START Program of the Faculty of Medicine (126/16) and Exploratory Research Space (OPSF449), RWTH Aachen.

*Conflict of interest.* None declared.
